# Drivers Shaping Spillover of Aleutian Mink Disease Virus Introduced With American Mink Among Native Mustelids

**DOI:** 10.1155/tbed/3184679

**Published:** 2025-02-23

**Authors:** Andrzej Zalewski, Marta Kołodziej-Sobocińska, Jenni M. E. Virtanen, Hanna Zalewska, Tarja Sironen, Karol Zub, Marek Nieoczym, Marcin Popiołek, Anna Wereszczuk

**Affiliations:** ^1^Mammal Research Institute, Polish Academy of Sciences, Białowieża 17-230, Poland; ^2^Department of Veterinary Biosciences, Faculty of Veterinary Medicine, University of Helsinki, Agnes Sjöbergin Katu 2, Helsinki 00790, Finland; ^3^Department of Virology, Faculty of Medicine, University of Helsinki, Haartmaninkatu 3, Helsinki 00290, Finland; ^4^Department of Zoology and Animal Ecology, University of Life Sciences, Akademicka 13, Lublin 20-950, Poland; ^5^Department of Parasitology, University of Wrocław, Przybyszewskiego 63, Wrocław 51-148, Poland

## Abstract

Invasive alien species pose a major threat to ecosystems by outcompeting native species for resources, altering habitats, enabling potential genetic hybridisation and introducing pathogens into the environment. An understanding of the factors that determine virus transfer between invasive and native species is crucial to the mitigation of the negative impact of the pathogens introduced. This study presents a comprehensive analysis of factors influencing Aleutian mink disease virus (AMDV) infection in native mustelids in Poland, following its introduction by feral American mink. AMDV seroprevalence in American mink varied spatially from 0 in the central and southern regions to 0.8 in the northern regions. Antibodies to AMDV were detected in all six studied mustelids, including a novel finding in weasels. AMDV seroprevalence in other mustelids correlated positively with its occurrence in American mink, and reached 0.54 in areas with the highest mink AMDV seroprevalence. Furthermore, in native mustelids, more closely phylogenetically related to mink, AMDV seroprevalence was higher (0.68 in polecats and weasels) compared to more distantly related species (0.37 in badgers). Over the 27-year study period, AMDV seroprevalence in mustelids has increased from 0.04 to 0.60, despite a decline in seroprevalence in feral mink in subsequent years. These findings suggest that the spread of viral infections as a result of the introduction of invasive species could affect mustelid species and may intensify over time.

## 1. Introduction

Current mass species extinction, with its impact on biodiversity loss, represents a critical global crisis that threatens the intricate web of life on the earth [1]. This loss of biodiversity not only erases the functional uniqueness of each species, but may undermine the functioning and resilience of ecosystems [2, 3]. Over the past century, human activities, such as overexploitation of natural resources, habitat destruction, the introduction of invasive species and pollution, have accelerated the rate of species threats and/or extinctions to unprecedented levels [4, 5]. The introduction of alien species to a new ecosystem is one of the important drivers of recent extinctions, with various devastating consequences for native fauna [6]. Invasive alien species often outcompete native species for resources, as well as posing a threat of genetic hybridization with native species and the introduction of a range pathogens (bacteria, viruses, or parasites) that may spill over to native species [7–13]. Some examples have shown the devastating impact of introduced pathogens on native hosts, for example, the malaria parasite *Plasmodium relictum* leading to the decline and extinction of native bird populations on the Hawaiian Islands [14] or the nematode *Anguillicola crassus* causing mass mortality in the eel *Anguilla anguilla* in Europe [15]. Along with viruses, which are known to cause rapid and high rates of animal mortality, invasive species also serve as vectors for pathogens that cause significant diseases, albeit not resulting in mass animal die-offs within a short period. Although the effects of these viruses in their introduced region were likely more diffuse over time and/or less studied, they may have had a significant detrimental effect on a wide range of animal species.

To initiate cross-species transmission, a virus must first be exposed to a new (native) host and successfully replicate within it [16, 17]. The rate of exposure is largely related to the ecological and behavioural traits of invasive and native host species [16]. It is also linked to the number of introductions (‘primary' cases of infection), which depend on the density and/or prevalence of infection in the invasive host [16]. A higher density of invasive hosts and high virus prevalence increase the probability of virus transmission. Therefore, the expansion of invasive species and the increase in their density after colonisation [18] may heighten the probability of virus exposure to native species over time. The rate of exposure may also be modified by other factors, for example, pathogen transition routes (e.g., by direct or indirect contact) or habitat niche overlap between the invasive and native host [16].

In the second step of cross-species transmission, the virus infects the native species, which is based on ‘compatibility' between the virus and the native species. The susceptibility of a new host to a new pathogen is related to various factors, with phylogenetic distance between hosts playing a significant role [19]. Generally, a virus will easily replicate in a new host that is closely related to the source host, given that, for example, the new host's cells will typically possess compatible cell receptors [20]. These receptors are often conserved across closely related species. This relationship is complex, involving factors such as host specificity, coevolution, host immune responses and viral evolution [19, 21]. Therefore, in the wild, some viruses may infect a broad range of host species from various groups. For example, avian influenza (H5N1) may infect various bird species as well as mammals [22, 23]. Conversely, certain viruses demonstrate high levels of host specificity, infecting only a particular species and/or a potentially closely related group of species (e.g., the squirrelpox virus) [24]. The final third step is the successful transmission of viruses between native hosts, which relies on the ecology, behaviour and genetic variability of the new hosts [16]. Taking all three steps into consideration, the prevalence of virus infections in native species introduced by invasive species is primarily influenced by the phylogenetic distance between species, the prevalence of pathogens and the density and spatial expansion of the invasive species. As the prevalence of pathogens and density of invasive species typically increase over time, virus transmission to native hosts may also increase, although such findings are rarely documented.

The American mink (*Neogale vison*), native to North America, is an example of a highly invasive species in Europe, Asia and South America [25]. Introduced to Europe and Asia in the early 20th century for fur farming purposes, American mink populations began to proliferate following the release or escape of some individuals into the wild, particularly in Russia. This led to the formation of feral populations around 1930 [25]. Their further rapid spread across European waterways has had a significant negative impact on native prey populations (e.g., birds and rodents) and their competitors [11, 26, 27]. American mink were introduced to Poland towards the end of the 1970s, in the north of the country. Since then, the mink has colonised almost the whole of Poland, except for its southernmost regions [18]. In their new range, American mink have inhabited various riparian habitats, including the banks of lakes, rivers, streams, midfields and fish ponds, but they have avoided urbanised areas [28, 29]. The mink density index, estimated as the number of mink captured per 100 trap-nights, increased to very high (9 mink/100 trap-nights) 10–15 years after local populations were established. In the following years, it reduced and stabilised at a level of ca. 5 mink/100 trap-nights [18].

The introduction of the American mink was accompanied by the introduction of the Aleutian mink disease virus (AMDV), a highly contagious parvovirus belonging to the species *Carnivore amdoparvovirus* 1 [30]. The virus can lead to Aleutian mink disease (AMD), a severe progressive disease with multiple clinical syndromes in American mink [31]. Some animals can clear the virus, while others experience chronic infection [32]. Infected feral mink experience a deterioration in body condition and some organs, particularly the liver and spleen, become enlarged [33]. Infected feral female mink have smaller litters, and the survival rate of their pups is lower compared to uninfected females [34]. AMDV prevalence in feral mink varies widely in both space and time [33]. Both high mink density and high AMDV prevalence greatly increase the exposure rate to native species. In Europe, there are nine native mustelid species, six of which are the most prevalent. The habitats of five (European mink *Mustela lutreola*, Eurasian otter *Lutra lutra*, European polecat *Mustela putorius*, stoat *Mustela erminea* and weasel *Mustela nivalis*) largely overlap with those of the American mink [35].

Infected American mink shed the virus in their bodily fluids, including urine, faeces and saliva [36, 37], which can contaminate the environment. Consequently, the spill-over of AMDV from feral American mink to native mustelids can occur through direct contact or indirect transmission via contaminated environments. Antibodies against AMDV or viral DNA have been found in several carnivores, including species from the genera *Mustela*, *Martes*, *Mephitis*, *Lutra*, *Genetta*, *Procyon*, *Lynx* and *Vulpes* [13, 38–42]. In some of these species, disease symptoms resembling those observed in American mink have been described [43, 44]. However, data and analyses of factors influencing the prevalence of AMDV in species other than American mink are limited. The present study aimed to analyse factors affecting the seroprevalence of AMDV antibodies in six species of native mustelids across a broad spatial scale. We hypothesise that: (1) the seroprevalence of AMDV in native mustelids is positively correlated with the seroprevalence of the virus in invasive American mink (exposure hypothesis); (2) AMDV seroprevalence in native mustelids increases over time with the spatial and demographic expansion of American mink and (3) AMDV seroprevalence is negatively correlated with phylogenetic distance of the particular native species to the American mink (‘compatibility' hypothesis). We initially estimated the variation of AMDV infection probability in American mink across a broad spatial scale in Poland. Next, we use these estimates of AMDV infection probability as well as the year of native mustelid sample collection and phylogenetic distance between mink to analyse AMDV infection in native mustelids.

## 2. Material and Methods

### 2.1. Mustelid Collection

**Figure 1:**
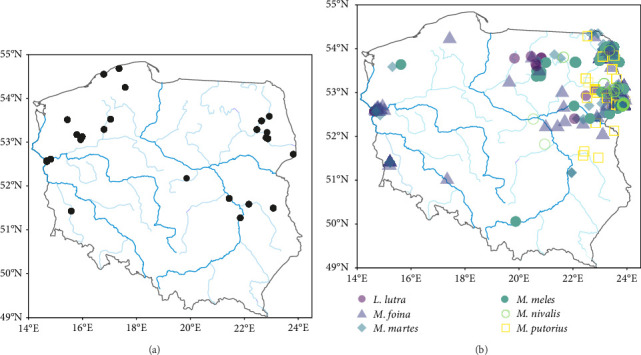
The locations of American mink trapping sites (A) and the locations of the collected carcases of six native mustelids (B) in Poland. Blue lines indicate rivers; darker lines indicate the four main rivers in Poland (Vistula, Bug, Oder and Warta); lighter lines indicate medium-sized rivers.

Between 1995 and 2022, we collected carcases of 1303 feral American mink and 446 mustelids of six species (weasel–38, polecat–31, pine marten *Martes martes*–125, stone marten *M. foina*–134, otter–14 and badger *Meles meles*–104). From the mustelid guild, we did not include the stoat in our analysis due to the population decline of this species in Central Europe (including Poland) following the invasion of the American mink [45]. American mink were collected from 25 trapping sites (Figure 1A), where feral mink were being eradicated to implement nature protection plans within bird conservation projects, under permission granted by local and government authorities and some mink, were collected as roadkill found near these trapping sites. Native mustelids, were opportunistically collected as roadkill or culled during legal hunts at various locations across Poland with only one individual of a particular species being found at each location. These locations were too distant from each other to be grouped into distinct sites, and therefore, we distinguish them from the mink trapping sites by referring to them as ‘locations' (Figure 1B). No animals were killed specifically for this study. Mustelid carcases were frozen and stored at −20°C before dissection. The samples collected were divided into two seasons: non-breeding (September–January) and breeding (February–August).

### 2.2. Serological Detection of AMDV Antibodies

The spleen and heart were removed during dissection, and filter paper strips, air-dried and kept at −20°C, were used to absorb blood from these organs. AMDV VP2 ELISA was employed to screen animals for AMDV antibodies, using a circular piece (5 mm) of filter paper that was then treated overnight in a 100 µL of dilution buffer (PBS + 0.5% BSA + 0.05% Tween 20) [46]. AffiniPure Goat Anti-Cat IgG (H + L; Jackson ImmunoResearch) peroxidase-conjugated at a dilution of 1:4500, or goat anti-ferret IgG (H + L) secondary antibody (Novus) at a dilution of 1:20,000, were utilised as conjugates. The ELISA cut-offs were determined separately for each antigen batch and conjugate by testing a panel of 10 negative samples in seven duplicates and adding two standard deviations to the mean absorbance. For more details, see Zalewski et al. [33] and Virtanen et al. [13].

### 2.3. Statistical Analyses

First, we estimated the spatial pattern of AMDV infection probability in American mink by performing a generalised additive model (GAM; package mgcv) with a binomial response variable. In a previous paper [33], we analysed the temporal and spatial (in three regions) variability of AMDV seroprevalence. In this article, we have not only expanded the sample size but also extended the spatial distribution of the surveyed sites to southern Poland. This provides the opportunity to predict the spatial variability of AMDV seroprevalence over large areas of Poland. To account for temporal and spatial variation in AMDV prevalence in mink, we included year in the GAM (with *k* = 15) and the interaction between longitude and latitude (with *k* = 25) as continuous explanatory variables, using thin plate regression splines. We also incorporated season and sex in the GAM as categorical explanatory variables. We generated a contour plot map of the predicted effects of AMDV infection probability in American mink from the model using the ‘vis.gam' function, which allowed for control within the range of the original covariate values using too.far = 0.25 to avoid undue extrapolation. Then, we assigned a predicted AMDV seroprevalence of American mink to each location of native mustelids.

We analysed the probability of AMDV infection in the native mustelids using a generalised linear model (GLM) with a binomial family in response to five factors: (1) the estimated probability of AMDV infection in American mink, predicted from the GAM (see description above) for each native mustelid location; (2) the phylogenetic distance between native mustelid species and the American mink; (3) year; (4) season (breeding and non-breeding) and (5) sex of the specimen. Variance inflation factors (VIFs) indicated non-collinearity among all explanatory variables (VIFs < 3), and thus, were included in the models together. In three cases, mustelid samples were collected from locations where AMDV was not predicted for mink by our GAM. As there were no mink in these areas or their density was very low [18], we assumed the AMDV seroprevalence in mink to be 0. The phylogenetic distance between native mustelids and the American mink was calculated by determining the pairwise distances between pairs of tips from a phylogenetic tree. A novel time-scaled phylogeny of musteloids [47] was obtained using the ctpm package [48]. We checked for spatial autocorrelation in AMDV seroprevalence using Moran's I statistic in the ncf package [49]. All statistical analyses were performed in R 4.2.1 [50].

## 3. Results

### 3.1. AMDV Seroprevalence of American Mink

**Figure 2:**
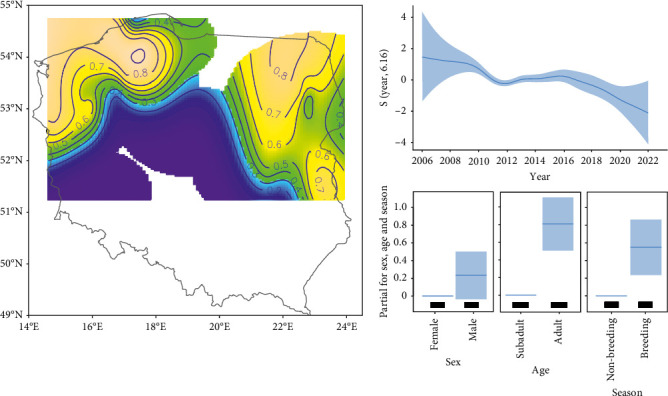
The predicted seroprevalence of Aleutian mink disease virus (AMDV) in American mink within its range in Poland and in relation to year, sex, age and season estimated from the generalised additive model (GAM). The shaded area denotes 95% confidence intervals (CIs).

Antibodies to AMDV were detected in 873 out of 1303 specimens (67.0%), across 22 out of 25 study sites. The GAM revealed a non-linear variation in AMDV seroprevalence in feral mink across the study area, with the north-east and north-west of Poland recording the highest seroprevalence (around 0.8–0.9; Figure 2). Conversely, the southern region of Poland exhibited the lowest probability of infection. Over the study period, AMDV seroprevalence decreased from 0.957 (confidence interval (CI) 95% = 0.952–0.962) in 2006 to 0.600 (CI 95% = 0.536–0.660) in 2020. Furthermore, compared to the non-breeding season (0.761, CI 95% = 0.737–0.783), AMDV seroprevalence was higher during the breeding season (0.846, CI 95% = 0.833–0.858). There were no differences in seroprevalence between the sexes, but AMDV seroprevalence was higher in adults (0.846, CI 95% = 0.833–0.858) than in subadult individuals (0.710, CI 95% = 0.679–0.740; Table 1, Figure 2).

### 3.2. AMDV Seroprevalence of Native Mustelids

The prevalence of antibodies against AMDV was lower in native mustelids compared to mink and reached 28% (125 AMDV-positive individuals out of 446) across all species. Seroprevalence was highest in the polecat (55%, 17/31), moderate in the stone marten (39%, 53/134), weasel (26%, 10/38), pine marten (25%, 31/125) and otter (21%, 3/14) and lowest in the badger (11%, 11/104).

**Figure 3:**
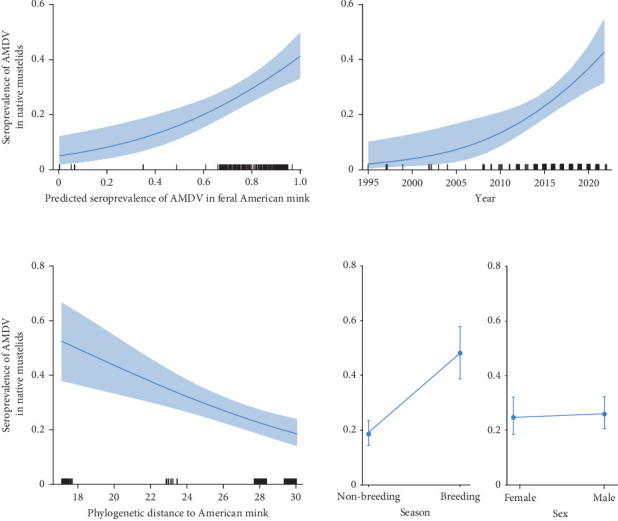
The predicted seroprevalence of Aleutian mink disease virus (AMDV) in six native mustelids in response to predicted seroprevalence in American mink in each native mustelids sample location (estimated from the generalised additive model (GAM)), consecutive years, phylogenetic distance of native mustelid to American mink, season and sex estimated from the generalised linear model (GLM). Curves and dots represent the estimated prediction, and shading or bars denotes the 95% confidence intervals (CIs).

The GLM revealed a rise in AMDV seroprevalence as the predicted seroprevalence in feral American mink increased. This ranged from 0 in areas with little or no mink infection to 0.540 (CI 95% = 0.513–0.567) in areas where the AMDV seroprevalence in American mink increased to 0.8 (Table 2 and Figure 3). A rise in AMDV seroprevalence was also observed over time, with values ranging from 0.040 (CI 95% = 0.037–0.042) in 1995 to 0.604 (CI 95% = 0.568–0.638) in 2022. Thus, within 27 years, AMDV seroprevalence in mustelids increased 15 times. As a species' evolutionary distance from the American mink increased, the probability of contracting AMDV infection decreased (Figure 3). Closely related species exhibited higher AMDV seroprevalence (0.682; CI 95% = 0.649–0.713) than more distantly related species (0.323; CI 95% = 0.301–0.346). AMDV seroprevalence in native mustelids was also significantly higher in the breeding season than in the non-breeding season, but there was no difference between sexes (Table 2 and Figure 3).

## 4. Discussion

This study provides a comprehensive analysis of factors influencing AMDV infection in mustelids, revealing significant spatial variation in AMDV seroprevalence among feral American mink populations in Poland. Moreover, it demonstrates that the six studied native mustelid species were infected by the virus, with weasels being identified as infected for the first time, thus, expanding the list of AMDV-affected species. The seroprevalence of AMDV in mustelids was correlated with the virus' seroprevalence in invasive species and the phylogenetic relationship between native species and introduced mink. Despite the decrease in the seroprevalence of feral mink in consecutive years, AMDV seroprevalence in mustelids has continued to rise over time. These findings suggest that the spread of viral infection, which may escalate over time, could impact mustelid species due to the introduction of invasive species that carry viruses.

Our study was only based on seroprevalence. However, other *Amdoparvoviruses* may also be circulating in wild mustelids and their cross-reaction with AMDV in ELISA can not be excluded (e.g., *Skunk amdoparvovirus* (SKAV) and *Labrador amdoparvovirus* 1 and 2 (LaAV-1 and LaAV-2)) [38, 51]. In our previous study about wild mustelids in Poland [13], only AMDV was detected in stone martens, pine martens and polecats. Based on this observation, it is likely that most of the seropositive individuals representing these species were infected by AMDV. No other *Amdoparvoviruses* have been found to be circulating in wild mustelids in Central and Eastern Europe as of date. Due to the previous results which indicated that most of the seropositive stone martens, pine martens, polecats and otters were not PCR positive [13], excluding cross-reaction by other *Amdoparvoviruses* would be challenging. It should be noted, however, that many of the other *Amdoparvoviruses* have either been suggested or shown to cause clinical disease in their host, making them a potential threat as well.

Comparing factors influencing seroprevalence in both mink and native mustelids, we demonstrated that, in both cases, seroprevalence was higher during the breeding season than in the non-breeding season. This may be due to increased contact between individuals during the breeding season, as well as larger home range sizes and individuals travelling longer distances [52]. Autumn dispersion of subadults likely plays a lesser role in virus circulation, as they are less frequently infected than adults in Poland, similar to other areas [53–55]. There was no difference in seroprevalence between sexes, neither in mink nor in native mustelids, consistent with other studies [39, 42, 53, 55]. However, this contrasts with our previous analyses, where females were less frequently infected than males [33]. These findings suggest that similar biological factors influenced the circulation of AMDV in populations of American mink and native mustelids.

This data reveals significant spatial variation in AMDV seroprevalence in American mink across Poland, generally with higher rates in northern regions and lower rates in the south. Similar substantial spatial variations in AMDV seroprevalence in American mink have been documented in Sweden (ranging from 38% to 71%), Spain (6%–47%) and Canada (Ontario; 0%–82%) [54–56]. The seroprevalence of AMDV is influenced by various factors, primarily the number of mink farms in the study areas [33, 56]. Additionally, our data suggests that the seroprevalence of AMDV in mink is lower in recently colonised areas, such as central and southern Poland. A key finding of our study is that the probability of AMDV infection in native mustelids is explained by the AMDV seroprevalence in American mink in a given area; an increase in seroprevalence in mink corresponds to an increase in native mustelids (exposure hypothesis). Although this correlation was expected, our study confirms this assumption for the first time. Moreover, on average, the seroprevalence in all six species was two times lower than in American mink. This aligns with similar observations of overall lower AMDV seroprevalence in other mustelids compared to American mink in previous studies [38–40, 42]. These findings imply that mink serve as the main host of AMDV, allowing it to persist in the environment and act as a source of virus transmission for other vulnerable species. The native species may function as partial reservoirs, potentially increasing the persistence of the virus.

The seroprevalence of AMDV in native mustelids was also associated with the phylogenetic distance of these species from the American mink (‘compatibility' hypothesis). Therefore, *Mustela* species, which are closely related to mink, exhibited higher seroprevalence, while the lowest seroprevalence was observed in the distantly related badger. In Spain, AMDV seroprevalence in American and European mink was similarly high (32%) [55]. A very high AMDV seroprevalence was detected in the stoat (71%), surpassing that of any other *Mustela* species [39]. Conversely, there has been very little or no evidence of infection in phylogenetically distant mustelids or viverrids, such as the North American river otter (*Lontra canadensis*), genet (*Genetta genetta*) [39, 40, 57] or in canids [38, 39]. Nonetheless, because AMDV infection is influenced by several factors, a wide range of the virus' prevalence in mustelids has been reported in the literature. For instance, higher infection rates in the stone marten than predicted based on phylogenetic relations [13] may be attributed to the species' predominance in rural areas, where mink farms are located, thereby, increasing the risk of infection [58]. On the other hand, there is no evidence of infection in pine martens or fishers (*Pekania pennanti*) in some areas where infected mink were present [39, 42]. The overlap of mustelids and mink niches, particularly habitat niches, as well as the territorial behaviour of mustelids, are other factors that may potentially modify this relationship. To determine the possibility of virus circulation in the animal community, more thorough evaluations incorporating a variety of parameters are required.

The AMDV-infected feral American mink had a larger liver and spleen, poorer body condition and females produced smaller litter with lower pup survival rates [33, 34]. However, there is conflicting evidence regarding the detrimental effects of AMDV on other mustelids. It appears that the virus cannot reproduce in native mustelids as effectively as in American mink, given that the percentage of native mustelids was positive for antibodies that were also PCR positive was substantially lower (10%–20%) compared to feral mink (96%) [13]. Additionally, evidence suggesting that the virus is not or is only mildly harmful to polecats and ferrets (*M. putorius furo*) arises from the fact that these animals did not exhibit elevated levels of gamma globulin, as often observed in infected AMDV mink [40, 44]. However, it is worth noting that experiments with infected ferrets were conducted for only 180 days [44]. In ferrets kept as pets for a longer duration, clinical signs of disease were observed in 43% of AMDV antibody-positive individuals [59]. The challenge in interpreting these data lies in the fact that AMDV strains in mink and ferrets are genetically different, yet both can infect ferrets [44]. Finally, elevated gamma globulin levels were observed in infected European mink, higher than in uninfected individuals and at similar levels to infected American mink [40], suggesting that these species may experience similar disease symptoms. These findings point to a lesser negative impact of AMDV on other mustelids than on American mink, especially those distantly related to the American mink and support the hypothesis that virus spill-over to more distantly related species leads to relatively milder disease [60]. However, virus infection may still reduce the fitness of at least some proportion of infected individuals in native mustelid populations, especially in species closely related to the American mink. In Spain, the seroprevalence of AMDV in European mink has increased over time [55]. This has led to suggestions that AMDV infection might contribute to the reported decrease in European mink populations in Europe [40].

## 5. Conclusions

Our research demonstrated that invasive species introduced with pathogens transmit them to native species, posing a major threat to the native species, particularly when closely phylogenetically related. While the introduction of pathogens may not always lead to a rapid and substantial decline in the number of native species, it could have a gradual impact as virus prevalence in native species rises over time. As a result, a longer time frame may be required to assess the effects of virus spill-over caused by invasive species. The lack of integration of a range of factors into a comprehensive prognostic framework remains the greatest challenge in understanding the circulation of introduced viruses and protecting native species. Integration research within the One Health concept is essential for management to minimise the emergence of infectious diseases into the environment and to reduce their negative impact on native species.

## Figures and Tables

**Table 1:** Summary of generalised additive model (GAM) estimating probability of Aleutian mink disease virus (AMDV) presence in American mink in relation to interaction between latitude (N) and longitude (E), year (2006–2022), season (breeding and non-breeding), mink sex and age (subadult and adult).

Variables	Estimate	SE	*Z* value	*p*
Intercept	1.55	0.17	8.93	>0.001
Sex (male)	0.23	0.13	1.73	0.083
Season (breeding)	0.55	0.16	−3.50	>0.001
Age (adult)	0.81	0.15	−5.37	>0.001

	**edf**	**Ref df**	***χ* ^2^**	***p***

s (N, E)	17.35	19.99	117.9	>0.001
s (year)	6.16	7.63	29.1	>0.001

**Table 2:** Summary of generalised linear model (GLM) analysis of the probability of Aleutian mink disease virus (AMDV) presence in native mustelids in response to predicted seroprevalence in American mink (mink seroprevalence), the year, phylogenetic distance of native mustelid to American mink, season and sex.

Variables	Estimate	SE	*Z* value	*p*
Intercept	−268.10	80.13	−3.35	0.001
Mink seroprevalence	2.63	0.60	4.38	<0.001
Year	0.14	0.04	3.35	0.001
Season (non-breeding)	−1.42	0.26	−5.46	<0.001
Sex (male)	0.06	0.24	0.25	0.80
Phylogenetic distance to American mink	−0.12	0.03	−4.02	<0.001

## Data Availability

The data that support the findings of this study are available from the corresponding author upon reasonable request.
